# Female Sexual Dysfunctions and Urogynecological Complaints: A Narrative Review

**DOI:** 10.3390/medicina58080981

**Published:** 2022-07-23

**Authors:** Lavinia Mosca, Gaetano Riemma, Andrea Braga, Matteo Frigerio, Alessandro Ferdinando Ruffolo, Mattia Dominoni, Gaetano Maria Munno, Stefano Uccella, Maurizio Serati, Antonio Raffone, Stefano Salvatore, Marco Torella

**Affiliations:** 1 Obstetrics and Gynecology Unit, Department of Woman, Child and General and Specialized Surgery, University of Campania “Luigi Vanvitelli”, 80128 Naples, Italy; laviniamosca@aol.it (L.M.); gaetano.riemma@unicampania.it (G.R.); gmm9401@gmail.com (G.M.M.); 2 Department of Obstetrics and Gynecology, EOC-Beata Vergine Hospital, 6850 Mendrisio, Switzerland; andybraga@libero.it; 3 Department of Obstetrics and Gynecology, ASST Monza, Ospedale San Gerardo, 20900 Monza, Italy; frigerio86@gmail.com; 4 Obstetrics and Gynecology Unit, Vita-Salute University and IRCCS San Raffaele Hospital, 20132 Milan, Italy; alesruffolo@gmail.com (A.F.R.); salvatore.stefano@hsr.it (S.S.); 5 Department of Obstetrics and Gynecology, Fondazione IRCCS Policlinico San Matteo, 27100 Pavia, Italy; matti.domino@gmail.com; 6 Department of Clinical, Surgical, Diagnostic and Paediatric Sciences, University of Pavia, 27100 Pavia, Italy; 7 Department of Obstetrics and Gynecology, AOUI Verona, University of Verona, 37100 Verona, Italy; stefano.uccella@univr.it; 8 Department of Obstetrics and Gynecology, Del Ponte Hospital, University of Insubria, 21100 Varese, Italy; mauserati@hotmail.com; 9 Division of Gynaecology and Human Reproduction Physiopathology, Department of Medical and Surgical Sciences, IRCCS Azienda Ospedaliero-Universitaria di Bologna, S. Orsola Hospital, University of Bologna, Via Massarenti 13, 40138 Bologna, Italy; anton.raffone@gmail.com; 10 Gynecology and Obstetrics Unit, Department of Neuroscience, Reproductive Sciences and Dentistry, School of Medicine, University of Naples Federico II, 80100 Naples, Italy

**Keywords:** female sexual dysfunctions, pelvic floor disorders, vulvodynia, postpartum sexual dysfunctions

## Abstract

Female sexual dysfunctions represent a real widespread problem, usually faced from a psychological point of view; however, millions of women worldwide are impacted by pelvic floor dysfunction, personal shame and social taboos, however, continue to inhibit free conversation on the subject. Women’s quality of life is considerably improved by screening, diagnosing, and controlling urogenital and sexual issues. This review aims to provide a critical perspective of urogenital conditions and common disturbances in female sexual function associated with these issues. It also includes a discussion of postpartum pelvic dysfunction.

## 1. Introduction

Female sexual dysfunction (FSD) is a complex biopsychosocial phenomenon. Defined as the inability to have an orgasm during sexual intercourse, even though it rarely is dangerous for health, it can cause psychological damages, such as anxiety, depression, and inadequacy [1]. Although women’s sexual motivation is built on intimacy, sexual arousals are important and biological and psychological causes rules how these arousals were developed [2].

Providers must proactively question patients about the possible presence of FSD. When a sexual problem arises, determine the kind of FSD and advise them on the best treatment options [2]. There is no single treatment that is successful for all types of FSD. As sexual medicine has become more defined as a multidisciplinary field, sexual medicine societies have played a significant role in understanding FSD; experts across clinical, behavioral health, psychology, policy, and research arenas have defined and described the epidemiological data, diagnostic approach, risk factors, and treatment paradigms for FSDs. Screening, diagnosing, and controlling the symptoms of sexual discomfort can enhance a woman’s quality of life significantly [1,2]. Patients may benefit from a multimodal and multidisciplinary team approach to manage sexual pain. [3].

However, the origin and the enduring of sexual dysfunction often leads to the interaction of physical and psychological causes [3,4,5], and their division into two different groups based on organicity is not coherent with current research or with clinical approaches oriented to these conditions’ identification and treatment [4,5,6,7]. Therefore, the dichotomy of mind-body is overcome using a unified classification to reduce these conditions’ stigmatization and encourage their identification and correct treatment by different specialists, not only mental health specialists [8,9,10].

Diagnosis and evaluation of sexual health are complex and often incomplete. Sexual dysfunction includes hypoactive sexual desire problem, sexual aversion disorder, female sexual arousal disorder, lubrication issues, female orgasmic disorder, and pain disorders. [11]. While validated questionnaires such as the Female Sexual Function Index (FSFI) have been established as quantifiable measures of sexual dysfunction, they are often insufficient for diagnosis without evaluation of psychological distress [12]. Furthermore, studies often report total questionnaire scores without domain scores (e.g., desire, arousal, orgasm, and pain) [13].

This article reviews several urogenital conditions and common disturbances in female sexual function associated with these issues. It also includes a discussion of postpartum pelvic dysfunction.

## 2. Search Methodology

We performed an electronic literature search of the MEDLINE database (accessed via PubMed) for recent articles published from January 2012 to April 2022, in order to assess the role of FSD and urogynecological complaints. The following Medical Subject Headings (MeSH) terms were used to screen and identify studies: “Sexual Dysfunction” (unique ID: D012735) and “Urology” (unique ID: D014572) and “Female” (unique ID: D005260).

The selection criteria for this narrative review included: original articles (randomized and non-randomized clinical trials, including prospective observational studies, retrospective cohort studies, and case–control studies), review articles, and Cochrane analyses concerning pain relief for in-office hysteroscopy.

Articles that met the inclusion criteria were carefully read by three authors (L.M., G.R., and M.T.). If appropriate, adjunctive articles were extracted from reference lists and were also reviewed to insert other critical papers that could have been missed during the original search. A total of 177 references was thus used in this review. We present here a narrative synthesis of the available evidence about the topic.

## 3. Dyspareunia

A typical female sexual disease is dyspareunia, the pain felt during or after sexual intercourse [14,15]. The sexual response, according to Basson’s paradigm (2001), begins with sexual desire (spontaneous or not, externally or through cognitive motivation). Sexual arousal encompasses both subjective and physiological arousal, with little link between the two. The presence of sexual desire and sexual arousal are not sequential, although both are important to achieve sexual satisfaction [16].

As a result, the discomfort frequently influences other areas of the sexual intercourse, perhaps contributing to secondary female sexual excitement and orgasmic distress [16]. Dyspareunia can be superficial or deep. The superficial one is often due to a vaginal introitus inflammation, urogenital atrophy, a lubrification modification, vaginal or urinary infection, or vulvodynia; instead, the deep one can occur in women with Pained Bladder Syndrome (PBS) or with Interstitial Cystitis (IC), pelvic floor muscles spasms, uterine disease (such as deep endometriosis), ovarian, or intestinal [17]. In addition, any condition that causes dyspareunia can cause vaginismus [18], a muscular hypertone form of the pelvic floor connected to frequently or continuous spasms of the vagina and pelvic floor musculature that interfere with vaginal penetration and cause personal distress. Furthermore, this can contribute to secondary functional orgasmic upset and persuade women to avoid sexual intercourse [16]. In such scenario, endometriosis affects approximately 10% of reproductive-aged women, reduces sexual functioning, and increases pelvic pain. Dysmenorrhea, persistent pelvic pain, dyschezia, and profound dyspareunia are all kinds of pelvic discomfort that are linked to endometriosis [19]. About 50% of women with endometriosis have deep dyspareunia, which can lead to sexual dysfunction and have a detrimental effect on relationships [20]. Because deep dyspareunia is not always successfully treated with conventional hormonal and surgical treatments for endometriosis, a deeper knowledge of the pathophysiology and related variables that cause deep dyspareunia is required [19,20]. It has recently been suggested that other comorbid conditions (such as painful bladder syndrome), myofascial contributors, and central sensitization of the nervous system may be significant in the pathophysiology of deep dyspareunia in endometriosis, in addition to endometriosis-specific factors (e.g., stage, location, and depth of invasion) [19]. For instance, a retrospective investigation found a link between profound dyspareunia and bladder discomfort [21]. Additionally, in a prospective investigation, it was shown that painful bladder syndrome and levator ani tenderness, unrelated to tenderness at other anatomic locations, were associated with more severe deep dyspareunia [22]. The role of interstitial cystitis and stress urinary incontinence on dyspareunia are shown in the next sections of this review.

## 4. Interstitial Cystitis/Painful Bladder Syndrome (IC/PBS)

The current definition accepted by the American Urological Association (AUA) and the Society for Urodynamics and Female Urology (SUFU) is: “An unpleasant sensation (pain, pressure, discomfort) perceived to be related to the urinary bladder, associated with lower urinary tract symptoms of more than six weeks duration, in the absence of infection or other identifiable causes” [23]. Prevalence studies for IC/BPS have been challenging to conduct due to the variable presentation of the disease but are estimated to be between 0.83% and 6.5% for women [24,25]. In comparison to the general population, women with IC/BPS have been observed to have greater rates of dyspareunia, altered desire, reduced lubrication, and decreased orgasm frequency [26]. Sixty-one percent of women in the RAND Interstitial Cystitis Epidemiology (RICE) research reported problems with sexual arousal, and 64 percent indicated a lack of desire. Dyspareunia affects between 49 percent and 90 percent of patients and is thought to be caused by bladder contact during intercourse. According to the RICE study [27,28], is one of the primary causes of chronic pelvic pain and deep dyspareunia; the bladder could be the source of chronic pelvic pain in 85 percent of cases (we are talking about nine million women) [29]. The key symptoms are urinary urgency, nocturia and frequent daytime urination. The patients can also have only one sign initially and then develop all other symptoms over several years [30,31]. Often it is undiagnosed or under-diagnosed. Therefore, it is connected to depression, dyspareunia, sexual dysfunction, and quality of life decrease [32,33]. According to Butrick et al. [34], up to 75% of IC/PBS patients reported worsened symptoms following sexual activity. Up to 54% of women with IC/PBS avoid having sexual relations with their spouses “the most of the time” [35,36]. In individuals with IC/BPS, painful sexual activity was thought to be a reliable indicator of quality of life. It was shown that up to 54% of women with IC/BPS frequently avoid having sexual contact with their partners, and IC/BPS was found to be associated with sexual dysfunction [37]. In a different study looking at female sexual dysfunction (FSD) in IC/BPS, it was discovered that these conditions were linked to fear of pain and pelvic discomfort experienced during sexual activity in adolescence and maturity. The reported rates of fear of pain and pain during sexual activity in adolescence and adulthood were 13.9–39.8% and 50.2–67.2% vs. 8.3–21.1% and 13.4–18% (*p* = 0.018, *p* = 0.001, *p* = 0.001, and *p* = 0.001) substantially higher in IC/BPS patients than the controls [38]. Following IC/BPS diagnosis, a decrease in sexual desire and orgasmic frequency was also noted in IC/BPS patients [39,40]. When IC/BPS patients were compared to controls, the mean value of the FSD scale was found to be considerably higher (18.5 14.3 vs. 8.3 10.2, *p* = 0.001) [40]. A score of 15 or above on the FSD measure denotes sexuality-related discomfort [40].

To improve therapy results in these individuals, early detection is critical [41,42,43]. Early on in the illness, mild and intermittent symptoms tend to become regular and severe [30]. Furthermore, women with PBS/IC have much more hysterectomies and other pelvic procedures than age-matched controls [39], with the majority of them occurring prior to the diagnosis of PBS/IC. However, it is unclear whether these surgeries were undertaken due of misdiagnosed PBS/IC or because the surgery caused persistent pelvic discomfort in the first place. There are two kinds of questionnaires, IC/PBS validated, that try to help in differential diagnosis for other diseases; they are the Pelvic Pain and Urgency/Frequency Scale (PUF) and O’Leary-Sant IC (ICSI-ICPI) [44,45]. Patients with IC/PBS vary in the areas of their bodies that they describe as painful and inhibitory for sexual activity. In a survey of 565 patients with IC/PBS, 60.8% reported pain in the vulvovaginal area, 56.7% in the lower abdomen, and 53.2% in the suprapubic region [46]. Dyspareunia can result from any of the painful sites mentioned above but is particularly dramatic in patients with periosteal vestibular erythema and inflammation, characteristic of provoked vestibulodynia (a condition associated with IC/PBS in up to 60% of cases) [36].

Patients with dyspareunia and sexual dysfunction caused by IC/PBS are treated in a variety of ways. It should be tailored to treat all sources of pain at the same time (e.g., targeting the bladder, pelvic floor muscles, and the vulva). Dietary changes, timed voiding, oral pharmacologic therapy, bladder instillations, physical therapy, and sex therapy are frequently used in treatment [47]. Patients might obtain insight into their symptoms and find causes by maintaining a bladder diary (including certain foods, activities, or phases of their menstrual cycle). Elucidating the timing of the pain related to sexual activity (e.g., superficial touching, arousal, penetration, thrusting, orgasm, or post-coital activity) can help the physician tailor therapy [46]. Several aids are available: non-pharmacological therapies (Global Therapeutic Massage (GTM), Pelvic Floor Myofascial Physical Therapy (MPT)) for the treatment of women with newly symptomatic IC/BPS [48]; oral therapy with amitriptyline [49]; intravesical instillation therapies utilized hyaluronic acid or both combined hyaluronic acid/chondroitin sulfate or chondroitin sulfate alone. Unfortunately, no therapy method examined thus far has shown to ameliorate sexual dysfunction in the IC/BPS population. Although sexual dysfunction is more common in IC/BPS patients than in controls, it has been suggested that treating the condition alone may not be enough to address the multiple causes of sexual dysfunction [22].

## 5. High-Tone Pelvic Floor Dysfunction

Maintaining proper pelvic organ function necessitates the use of pelvic floor muscles. The coccygeus and elevator ani muscles in the pelvic diaphragm play an important role in sexual response. The surrounding visceral structures usually impact these muscles (bladder and rectum). Seventy percent of women with genitourinary, bowel, and female sexual problems have abnormal pelvic muscle function [50,51]. Vaginismus, tension myalgia of the pelvic floor, or levator ani syndrome [52,53] are all terms for high-tone pelvic floor dysfunction, which is linked to sexual dysfunction [54,55]. Traumatic vaginal birth, direct neuromuscular damage to the pelvic floor, persistent unconscious bracing behaviors (e.g., holding pee for hours), sexual abuse, severe anxiety disorders, spinal injury, postural stresses, microtrauma, and infection are all possible causes [56]. Pelvic pain syndromes, such as IC/PBS, vaginismus, vestibulodynia, persistent pelvic pain, irritable bowel syndrome, and endometriosis, are common urogynecologic contributors to hypertonic states [48]. Frequency, urgency, dysuria, urine retention, fecal retention, dyspareunia, and vaginismus are all indications of high-tone pelvic floor dysfunction [51,52,53,54,55,56,57,58].

Pelvic floor tone may be assessed using a digital exam to determine a woman’s capacity to isolate, contract, and relax her pelvic floor muscles [59]. The vaginal side lower walls are lightly pressed during the digital examination. Without tightening both abdominal groups, gluteus, or abductors, the patient must tighten the finger and lift the pelvic floor. If patients cannot produce strength enough to tighten or sustain the “grip” for 5 s or in case of pain/tension during the pressure, probably there is a spastic pelvic floor dysfunction [60]. These results can be verified using specific tools to measure muscular activity, such as a vaginal or anal manometer or electromyography [40,51,61]. Therapy is finalized to muscular rehabilitation to simplify comfort and sexual pleasure. The physical rehab therapy of pelvic floor muscles, using relaxing techniques, pelvic reinforcement and stabilization, biofeedback, and electrical stimulation, proves to be an efficient treatment for high tone pelvic floor dysfunction. Internal pelvic floor massage (Thiele massage) makes the administration of a vaginal expander to prepare for sexual penetration much easier [53,54,55,56,57,58]. Physical treatment [59] may also benefit from precoital analgesics, antispasmodics, and muscle relaxants [57]. Tender point release and/or trigger point injections are another biomechanical therapy for persistent pelvic pain and hypertonus of the pelvic floor muscles. These locations in the levator ani muscle, which can be located everywhere in the pelvic floor musculature, have been segregated for therapeutic intervention. Patients may benefit from intravaginal muscle relaxants, trigger point injections, or botulinum toxin injections in the pelvic floor muscles if physical therapy fails to reduce muscular soreness and tenderness [60].

## 6. Vulvodynia and Vestibulodynia

Provoked vestibulodynia, also known as vulvar vestibulitis syndrome, is a common cause of penetrative superficial dyspareunia. PVD (provoked localized vulvodynia) is a prevalent and devastating chronic vulvar pain disease that affects 7–13% of premenopausal women [61,62]. During touch, pressure, and attempted or achieved vaginal intercourse, women with PVD express an acute pain or burning feeling localized at the vaginal opening [63,64,65]. The pain does not only affect sexual function and satisfaction [66], but is also associated with psychological distress [67,68], relational dissatisfaction, and a lower quality of life [59]. Yet, it is a neglected pain condition [69], and many women are left without correct diagnosis and treatment [70]. Other typical symptoms include allodynia within the vulvar vestibule for at least 3 to 6 months and erythema at the inferior introital sulcus, medial to the Hart line, particularly in the periosteal areas of the vestibular glands [62,63]. The Skene glands and/or parts of the anterior fourchette may or may not be affected by hypersensitivity and erythema. [71,72]. Vestibulodynia has an etiology that is unknown. Women with vulvar vestibulitis syndrome are homozygous for allele 2 of the IL-1RA gene (IL1RN*2), a phenotype linked to ulcerative colitis, Crohn’s disease, and systemic lupus erythematosus, according to one explanation. Proinflammatory reactions are more protracted and intense in persons with this phenotype than in those with other IL-1RA genotypes [70]. Another notion is that unpleasant environmental stimuli cause neurogenic inflammation of the vulvar vestibule ”end-organ,” resulting in parasympathetic efferent and visceral nociceptive afferent hyperactivity. The body produces antidromic substance P and calcitonin gene-related peptides in response to this hyperactivity, with nitric oxide processing happening in the tissues around the introital glandular ostia [72].

Vestibulodynia is most commonly triggered by sexual activity, although it can also be triggered by constrictive clothes, the use of a tampon, or a pelvic examination [71,72,73]. The criteria standard in clinical use to measure the localized pain associated with vestibulodynia remains the simple “touch test.” [73]. A saline-moistened swab is used to firmly contact locations on the labia majora, interlabial sulci, and lateral labia minora to complete this test. The woman’s reaction to these touches is compared to her reaction to comparable “touches” delivered to the ostia of the Skene glands, as well as those of the major and minor vestibular glands. [74] When tissues such as the labia majora or perineum are stroked, a woman with vestibulodynia will report little or no discomfort, yet acute sensitivity when the gland openings are touched. Obtaining a fungal culture for species type is the best way to rule out coincident candidiasis. Encourage the patient to hold a mirror and look into the vestibule after the touch test to demonstrate the biological (rather than psychogenic) origin of her ailment and to acquaint her with genital anatomy [75,76,77,78]. To date, there is no “gold standard” treatment for women with vulvodynia [68]. Non-pharmacological as well as pharmacological and surgical treatments have been tested, both individually and in combination. Non-pharmacological options have primarily been variations of cognitive-behavioral therapy (CBT), pelvic floor physical therapy, and alternative therapies such as acupuncture and Transcutaneous Electrical Nerve Stimulator (TENS). Agents that target peripheral and central pain processes, muscle relaxants, and surgical procedures are among the pharmacological and medical choices [79]. Despite PVD being the most common type of vulvar discomfort in premenopausal women, most therapy trials have included heterogeneous samples, not just women with PVD. Current treatment recommendations are largely based on clinical experiences [80,81,82], and the literature review shows that there is no evidence of the superiority of one treatment over another. When compared to lidocaine treatment, multimodal physiotherapy was the only strategy where some evidence could be shown (with low certainty) [83].

## 7. Pelvic Floor Disorders

Pelvic Floor Disorders (PFD) such as Urinary Incontinence (UI), Fecal Incontinence (FI), and Pelvic Organ Prolapse (POP) impact women’s sexual life. PFD can have a profound effect on women’s social, sexual, psychological, and financial well-being, resulting in social isolation, loss of income, and poorer quality of life [84].

### 7.1. Pelvic Organ Prolapse

The aberrant descent or herniation of the pelvic organs from their normal position, resulting in an abnormal feeling or function, is characterized as POP [85]. A fall of 1 of the anterior compartments (cystocele), the posterior compartment (rectocele and enterocoele), the uterus (cervix), or the apex of the vaginal canal (after hysterectomy) can all be categorized as POP. The anterior compartment prolapse is the most common site for POP (34%), followed by the posterior compartment (19%), apical (14%), and multi-compartment POP (14%). The prevalence of prolapse is predicted to rise in Western populations as a result of an ageing population and increasing obesity [86,87,88]. Women have an 11% lifetime risk of undergoing surgical correction of POP, with the anterior compartment being the most common site for repair at 40% [13].

POP has a complicated and multifactorial pathophysiology. The primary risk factors for POP include parity, vaginal delivery, growing older, and BMI, and the preoperative stage is a risk factor for POP recurrence. The degree of symptoms and how they affect quality of life, the stage and kind of POP, the patient’s expectations, overall health, and any prior treatments all influence the sort of treatment that is utilized [88].

Symptoms in POP-afflicted women may not always correspond to the physical degree of prolapse. Depending on the degree of the symptoms, an increasing severity of POP is most frequently weakly to moderately connected with particular symptoms related to voiding, defecatory, or sexual dysfunction [89].

Due to the complexity of human sexual function and the numerous factors (both personal and environmental) that have a significant impact, it is challenging to analyze the impact of POP on sexual function [90]. The complicated nature of sexual function is presumably the cause of the contradictory findings reported in the literature [91]. Additionally, when evaluating sexual function in POP, the common correlation between POP and lower urinary tract problems, particularly urine incontinence, is a confusing issue [92]. It is not always obvious from the data whether POP or urine incontinence do more impairment to sexual function when there is a negative connection between pelvic floor problems and sexual function [88]. In women with POP, there is a significant positive correlation between sexual function and self-perceived body image [91]. POP is linked to unpleasant emotions including poor self-esteem, depressive sensations, and the perception of losing one’s beauty [88,89,90,91]. Therefore, any enhanced sexual function following POP therapy may be due to a more positive perspective of one’s physique [89,90]. Despite the majority of partners being comforting and not complaining, most women with POP feel less confidence about their partner’s sexual experience than women without POP [91].

The majority of the time, sexual function scores following POP surgery are either improved or remain stable. Despite these encouraging results, it is still challenging to predict the sexual outcome of surgery, and none of the surgical procedures used to treat POP are risk-free. Counseling for prolapse surgery and sexual function is increasingly becoming more and more recognized as a crucial element [92].

Sacrocolpopexy, whether carried out by laparotomy, laparoscopy, or robot-assisted laparoscopy, which is increasingly more common, reduces the risk of dyspareunia and is the preferred method for POP repair, especially for women under the age of 70 [92].

Whatever the impact of surgery on sexual results, the couple’s preoperative sexual health continues to be the most important indicator of postoperative sexual satisfaction. As a result, evaluation of preoperative sexual function is critical before any pelvic floor reconstruction and should entail a multidisciplinary approach, sometimes involving the advice of a psychologist and sex therapist [88,89,90,91,92]. Couples with poor preoperative sexual function and patients with pelvic and/or perineal discomfort or dyspareunia should be thought of as being at risk for poor postoperative sexual function. Treatment alternatives for these individuals should be reviewed, and a suitable, personalized treatment plan should be suggested based on the patient’s expectations [91,92].

### 7.2. Urinary Incontinence

The involuntary loss of urine is known as UI. It can lead to issues with sexual female life, such as urine loss during coitus (coitus incontinence), Urge Incontinence (UUI)/Overactive Bladder (OAB)-Wet Syndrome [93], night losses associated with urgency, and bedwetting phobia [94,95]. Fear of odorous and urinary incontinence during coitus is linked to a change in image and self-esteem, which contributes to incontinent women’s low sexual activity frequency [96]. Urinary incontinence has a negative impact on sexuality in the older population when there is a sexual partner present [97]. Urinary incontinence linked to coitus has been classified into two categories: urine incontinence linked to penetration and urinary incontinence linked to orgasm (“squirting”) [98]. Incontinence associated with penetration has been linked to Stress Urinary Incontinence (SUI) and is linked to possible sphincter intrinsic dysfunction, and it is more common in women with SUI, as shown by urodynamic examination [99]. Although these data are not completely clear, coitus urine incontinence accompanied with orgasm has been linked to detrusor overactivity [100,101]. Several studies looked into the link between various types of urine incontinence and sexuality. Many thinks UI is confined to the elderly; however, a population-based survey reported an overall prevalence of 17% of UI in women >20 years of age [102,103]. Regarding the different types of UI, SUI was 3.7% in women <39 years vs. 8.0% in women >60 years, I was 1.0% in women <39 years vs. 2.5% in women >60 years [104]. Obesity, chronic cough, smoking, and ageing are associated with SUI.

UI in women can have a sexual influence depending on a variety of factors, including physical, psychological, social, and cultural factors. Due to their frequent comorbidity with other pelvic floor illnesses including pelvic organ prolapse, which also influence sexual pleasure, evaluation of the effects of SUI and lower urinary tract symptoms on sexual function is frequently skewed [105]. The research, which demonstrates a significant variance in sexual functioning features in women with incontinence both before and after therapy, does indeed represent these complications. Heterogeneity in research design, quality, and analysis highlights this discrepancy even more [106]. The different SUI therapies, such as transvaginal tape operations, might have a favorable or negative impact on sexual function. The cure of coital incontinence, attained by >90% of women, largely explains the sexual improvements described in multiple investigations [107]. It appears to be a good predictor of an improvement in postoperative sexual parameters. In contrast, decreased sexual function has occasionally been seen following surgery, with de novo or worsening dyspareunia being the most frequent culprit. On the basis of the result of sexual function, there are no compelling justifications in the literature for either one treatment or the other [105,106,107].

The most prevalent kind of UI, OAB syndrome, has a significant negative impact on quality of life, particularly sexual life. Unfortunately, most women, especially those who already experience OAB symptoms, seldom express concerns about the decline in sexual function. It has been shown that patients with OAB score lower on the Female Sexual Function Index and Pelvic Organ Prolapse/Urinary Incontinence Sexual Questionnaire [108], indicating that their sexual function is impaired. Traditional pelvic floor physical therapy, anticholinergic medications, and onabotulinumtoxinA (commonly known as Botox) intravesical injections are the first line of treatment for OAB. Last but not least, sacral neuromodulation is the preferred treatment for individuals who have rejected conventional treatments [109].

Mixed Urinary Incontinence (MUI) is a complaint of involuntary urine leakage that is linked to both urgency and effort or physical exertion, as well as on sneezing or coughing [110].

A recent study reported that, in comparison to women with UUI or SUI, individuals with MUI showed much higher levels of quality-of-life dissatisfaction, significantly lower total King’s Health Questionnaire ratings due to the overall negative effects of UI on quality of life, and significantly greater physical restrictions [111]. Women with MUI had a poorer quality of life than those with SUI and UUI, according to other research. Additionally, participants’ scores on the WHOQOL-BREF scale for their overall score, social relationships, environment, and psychological health were worse [111].

### 7.3. Anal Incontinence

Anal incontinence (AI) is defined as the involuntary loss of flatus or of feces (FI), which can be solid or liquid, whereas coital FI occurs with fecal leakage during vaginal intercourse [85]. Two population-based studies show a lifetime prevalence of 8.9–9.4% for FI [112,113], which increases with age, with 15.3% of women aged 70 experiencing monthly episodes of FI [114]. Childbirth trauma resulting in anal sphincter damage is the major cause of FI in women, whereas instrumental delivery, obesity, diarrhea, and the presence of multiple comorbidities also have a role [112,113].

For many women, AI is a devastating symptom associated with shame, embarrassment, and social isolation [114,115,116,117,118,119,120,121]. Studies looking at the effect of AI on sexual function are limited; however, women with AI were found to have decreased sexual desire, sexual satisfaction, arousal lubrication, and orgasm compared with those without [122,123,124,125,126]. Fear of soiling during intercourse, embarrassment and dyspareunia were the major impactors on sexual function. Women with AI report higher rates of FSD compared with those with UI, although the rate of sexual activity was similar. A reasonable explanation is that women suffering from this condition adopt coping strategies to reduce the impact of AI on sexual function [127,128]. There is little evidence that Pelvic Floor Muscle Therapy (PFMT) improves sexual function. In women with UI, PFMT has been demonstrated to improve sexual desire, performance during coitus, and the ability to attain orgasm while having no effect on arousal [129,130]. In women with SUI, PFMT has been shown to minimize incontinence and Increase sexual satisfaction [131].

## 8. Postpartum Sexual Dysfunction

One of the central points of this review was to analyze how childbirth impacts women’s sexual life. Sexual function declines during pregnancy and does not return to its baseline levels in the postpartum. This is in part attributed to changes in body image, lack of sleep, tension, urinary stress, and urge complaints [132,133]. Despite the significant impact on quality of life, the sexual function of women after childbirth is often neglected by health professionals [134].

### 8.1. Modalities of Childbirth

Leaving aside the psychological and social effects linked to the birth event, we have observed that vaginal birth involving anatomical and functional alterations in the muscles of the pelvic floor can cause sexual problems with a decrease in quality of life [135,136,137]. After childbirth, there is a significant worsening in all sexual domains, with dyspareunia, lack of vaginal lubrication, difficulty in reaching orgasm, vaginal bleeding or irritation after sexual activity, and loss of desire [138,139,140,141,142,143,144,145,146,147,148]. 

Eighty-nine percent of women resume sexual activity within six months of having delivery, according to studies. Prevalence rates of sexual dysfunction range from 41% to 83 percent at 2 and 3 months postpartum [135,136,137,138,139,140] to 64% at 6 months postpartum [125], but do not approach pre-pregnancy levels of 38% [148]. According to several research, women report significantly reduced levels of sexual pleasure and emotional satisfaction after 18 months [149,150,151,152,153,154,155]. Both spontaneous and aided vaginal deliveries have been linked to short-term perineum injury and long-term pelvic floor alterations. Many research projects have attempted to determine whether or not these alterations are to blame for the altered sexual function [150,151,152,153,154,155,156,157,158,159,160]. The data in the literature is debatable. Only a few studies have found a link between vaginal birth and sexual dysfunction [150,156]. Griffith et al. [156] evaluated 208 women at two years postpartum and reported a significant reduction in sexual satisfaction in women who had undergone vaginal delivery compared to women who had undergone elective caesarean section after a two-year follow-up. Compared to women who had a caesarean delivery, there was a substantial increase among the prevalence of urine incontinence, flatus incontinence, dyspareunia, and subjective depression in women who had a vaginal delivery [156]. Another study evaluated sexual function in 99 women, and found a significant increase in dyspareunia at three months in patients who had undergone vaginal delivery (45.3% vs. 11.9%; *p* < 0.001) [158].

Barret et al. [148] concluded that while dyspareunia at three months postpartum is significantly associated with vaginal delivery, at six months, there is no significant difference between vaginal and caesarean sections [124,135]. Van Brummen et al. [160] conducted a prospective study of 377 primiparas showing that sexual satisfaction at 12 months postpartum was not related to the mode of delivery. One year after childbirth, dissatisfaction with a sexual relationship was linked to not having been sexually active at 12 weeks of pregnancy and maternal age at delivery [157]. The majority of the prospective data obtained found that the manner of delivery has no meaningful impact on postpartum sexual function in the short and long term. Instead, it is possible that assisted vaginal delivery is associated with a worsening of sexual function. [158].

### 8.2. Parity

Sexual function is also affected by parity: primiparas have much greater dyspareunia than multiparas. The greater prevalence of severe perineal injuries and assisted delivery in primiparas might explain this [159,160,161].

### 8.3. Perineal Trauma

Severe perineal trauma, including third- and fourth-degree perineal lacerations, has an incidence of 1–3% in European countries and 2.2–19% in the USA [162]. A prospective study of 377 primiparas found that women with a grade 3 to 4 perineal tear were five times less likely to be sexually active than women with intact perineum due to more significant and longer-lasting dyspareunia [163]. Severe perineal trauma during labor is associated with decreased sexual function after delivery, with a higher incidence of dyspareunia and a longer time to resume sexual activity [148,164,165,166,167]. Grade 1–2 lacerations have no significant effect on perineal pain and dyspareunia in the postpartum period [168].

### 8.4. Episiotomy

Episiotomy, with rates ranging from 9.7% in Sweden to 100% in Taiwan [169], performed routinely, does not reduce sexual dysfunction [157]. There is no evidence that episiotomy enhances sexual function. At 1, 2, and 6 weeks postpartum, a prospective trial of 243 women reported no significant difference between vaginal birth with or without episiotomy [170].

### 8.5. Breastfeeding

Breastfeeding is of unquestionable benefit to maternal health [171]; however, due to low levels of estrogen, progesterone, and androgens, and high levels of prolactin indirectly impact on sexual interest by lowering vaginal lubrication, leading to atrophy, raised breast sensitivity, and lowered sexual desire [172,173]. Several studies have concluded that breastfeeding delays the resumption of sexual intercourse after childbirth [172,174,175]. Furthermore, the hormonal effects associated with breastfeeding appear to be associated with increased vaginal dryness and loss of libido [176,177].

## 9. Conclusions

The variety of problems associated with FSD is difficult to understand and identify. The figure of the gynecologist plays a key role in investigating the main organic pathologies that can also be the basis of psychological dysfunctions related to satisfaction. The knowledge of the main disorders of the pelvic floor and of all that is connected to dyspareunia, among the main causes of sexual discomfort, can help to orientate in the magnum sea of close interconnections between the various problems related to the sphere of sexuality.

From the studies reviewed, the basic problem is that we are dealing with an organic problem that, among other things, can cause sexual dysfunction, and therefore any therapy can lead to an improvement in sexual function only as a secondary effect. What is not addressed is the overall problem: it is now complex to carry out a study that focuses on FSD in all its complexity of facets and that takes on the burden of evaluating possible preventive, diagnostic, and therapeutic strategies in a multidisciplinary setting.

This review aimed to evaluate genito-urinary issues related to FSD. It was seen that these pathologies are often interconnected and are often the cause or consequence of psychological alterations underlying the dysfunctions. Perhaps for this reason, given that they are interconnected problems, therapy should also be evaluated on several fronts, not only on the purely biological one. We have herein summarized the most and less common urogynecological issues that could lead to FSD and how are these problems managed according to recent literature, in order to provide the women’s health clinician with the most up-to-date evidence on the complaint.

However, the central problem with current studies is the lack of consensus on the best treatment for sexual dysfunction in patients with genitourinary problems. In such scenario, the International Collaboration for Harmonising Outcomes, Research, and Standards in Urogynaecology and Women’s Health (i-CHORUS) is setting new standards in the development of protocols and guidelines to promote high-quality, low-bias standard of care on female pelvic floor disorders and other issues related to women’s health.

It is, therefore, believed that prospective studies carried out in tandem with other professional profiles are necessary so that the Cartesian dichotomy of organic and psychological components can be overcome even for the therapies offered.

## Data Availability

Not applicable.
